# Robotic Rehabilitation Using the Hybrid Assistive Limb for Drop Fingers in a Patient with Cervical Spondylotic Radiculopathy: A Case Report

**DOI:** 10.3390/jcm15135182

**Published:** 2026-07-02

**Authors:** Yuichiro Soma, Yukiyo Shimizu, Hideki Kadone, Shigeki Kubota, Yasushi Hada, Yasuhiro Homma, Masashi Yamazaki

**Affiliations:** 1 Department of Orthopaedic Surgery, Institute of Medicine, University of Tsukuba, Tsukuba 305-8575, Japan; skubota@tsukuba-seikei.jp (S.K.); masashiy@tsukuba-seikei.jp (M.Y.); 2 Division of Regenerative Medicine for Musculoskeletal System, Institute of Medicine, University of Tsukuba, Tsukuba 305-8575, Japan; 3 Department of Rehabilitation Medicine, Institute of Medicine, University of Tsukuba, Tsukuba 305-8575, Japany-hada@md.tsukuba.ac.jp (Y.H.); 4 Center for Innovating Medicine and Engineering (CIME), University of Tsukuba, Tsukuba 305-8575, Japan; kadone@md.tsukuba.ac.jp; 5 Department of Occupational Therapy, Ibaraki Prefectural University of Health Sciences, Ibaraki 300-0394, Japan; 6 Department of Orthopaedic Surgery, Ichihara Hospital, Tsukuba 300-3253, Japan

**Keywords:** Hybrid Assistive Limb (HAL), cervical spondylotic radiculopathy, drop finger, metacarpophalangeal joint, rehabilitation

## Abstract

**Background**: Drop finger may occur in patients with C7 and/or C8 cervical radiculopathy caused by cervical spondylosis. Although surgical decompression of the affected nerve roots is performed in patients with drop finger refractory to conservative treatment, postoperative recovery of drop finger is often unsatisfactory. Furthermore, no effective rehabilitation strategy for improving drop finger has yet been established. **Methods**: Here, we report a patient with drop finger who underwent a novel postoperative rehabilitation program. A 64-year-old man presented with drop finger of the left hand caused by left C7 and C8 radiculopathy and underwent cervical foraminotomy. For postoperative rehabilitation, we applied the single-joint Hybrid Assistive Limb (HAL), a wearable robotic suit. The patient underwent a total of 21 sessions of metacarpophalangeal HAL training, which assisted voluntary flexion and extension movements of the metacarpophalangeal joints, and 6 sessions of wrist abduction HAL training, which assisted ulnar-direction wrist abduction movements. **Results**: As a result, improvement in the left-sided drop finger was achieved. In this case, the use of HAL enabled voluntary motor training within the normal range of motion of the fingers and wrist even during the early postoperative phase, when sufficient neurological recovery had not yet been achieved. **Conclusions**: This successful motor experience may have facilitated the reacquisition of normal movement patterns, thereby contributing to improvement in drop finger.

## 1. Introduction

Drop finger associated with cervical spondylotic radiculopathy is caused by impairment of the C7 and/or C8 nerve roots [1,2,3,4,5,6]. Weakness of the finger extensor muscles results in difficulty extending the metacarpophalangeal (MP) joints. Surgical decompression of the affected nerve roots is performed in patients with drop finger refractory to conservative treatment; however, postoperative outcomes are not always satisfactory [7,8]. Furthermore, tendon transfer, nerve grafting, and nerve transfer procedures have been reported as surgical options for radial nerve palsy; however, functional outcomes remain variable and are highly dependent on the timing of intervention and the extent of reinnervation [9]. Conventional rehabilitation for drop finger mainly focuses on preventing secondary complications such as joint contracture, maintaining range of motion, and facilitating compensatory hand use through orthotic support and functional training [10,11,12]. However, these approaches do not directly target the selective recovery of finger extensor muscle strength or the restoration of fine motor control of the metacarpophalangeal joints, and evidence supporting interventions specifically aimed at improving drop finger function remains limited. The Hybrid Assistive Limb (HAL) is an exoskeleton-type robotic device for motion training support that was developed at our institution [13]. The therapeutic effects of HAL are considered to be mediated not only by simple power assistance, but also by repetitive motor learning based on the theory of interactive biofeedback (iBF), in which movements are initiated by bioelectrical signals derived from the neuromuscular system, thereby facilitating errorless motor learning [13]. Several types of HAL systems have been developed, including the bilateral leg HAL, single-leg HAL, single-joint HAL, and lumbar HAL, each of which is selectively used according to the clinical purpose. The single-joint HAL was initially developed for disorders involving the elbow and knee joints [14,15], and its clinical application has subsequently expanded to the treatment of shoulder and ankle joint disorders [16,17].

In the present study, we performed surgical treatment for a patient with left-sided drop finger and radial deviation of the wrist associated with cervical spondylotic radiculopathy. We developed a novel rehabilitation program using the single-joint HAL, consisting of metacarpophalangeal HAL (MP-HAL) training for voluntary flexion and extension movements of the MP joints and wrist abduction HAL (Wrist-Abd HAL) training for voluntary ulnar-direction wrist abduction movements. Voluntary motor training of the left hand was initiated during the early postoperative period. As a result, improvement in both the left-sided drop finger and radial deviation of the wrist was achieved.

## 2. Materials and Methods

### 2.1. Case Presentation

A 64-year-old man presented to our hospital with drop fingers of the left hand and neck pain as his chief complaints. Two weeks prior to presentation, he developed neck pain and left scapular pain upon awakening, followed by weakness of the left hand five days later, which resulted in drop fingers.

At the initial visit, in addition to drop fingers and neck pain, he exhibited sensory disturbance in the ulnar aspect of the left forearm and in the ring and little fingers of the left hand. Manual muscle testing (MMT) of the left upper extremity revealed decreased strength, with grades of 2 in the finger extensors, 4 in the finger flexors, 2 in the interossei, 4 in both the wrist extensors and flexors, and 4 in the triceps.

No abnormal findings were observed in the right upper extremity or in either lower extremity. Cervical spine radiographs showed no apparent abnormalities, and magnetic resonance imaging (MRI) of the cervical spine demonstrated no clear evidence of spinal cord compression.

Conservative treatment with a cervical collar was initiated, resulting in near-complete resolution of neck pain two weeks after the initial visit (four weeks after symptom onset). Muscle strength in the left upper extremity also showed a tendency toward recovery. However, drop fingers of the left hand did not improve thereafter. The dropping was particularly prominent in the ring and little fingers (Figure 1, arrow), and radial deviation of the wrist was also observed (Figure 1, arrowhead) (Video S1).

Myelographic examination revealed an osteophyte at the left C6–7 foramen (Figure 2b,c, arrowhead) and a space-occupying lesion suggestive of a disc herniation at the left C7–T1 foramen (Figure 2c, arrow). These findings suggested compression of the left C7 and C8 nerve roots.

Surgical intervention was performed three months after symptom onset. The procedure consisted of a posterior approach with left C6–7 foraminotomy (Figure 3a,b, arrowhead) and C7–T1 foraminotomy (Figure 3a,b, arrow). During the left C7–T1 foraminotomy, a disc herniation was identified within the foramen and subsequently removed.

Postoperatively, no improvement in drop fingers of the left hand was observed. At the patient’s request, rehabilitation using the HAL was initiated three days after surgery (Figure 4). In addition, conventional rehabilitation was performed in parallel with HAL training. During the inpatient period, occupational therapy was provided five times per week (weekdays), with each session lasting approximately 40 min. Conventional rehabilitation included passive range-of-motion exercises to prevent contractures and interventions aimed at maintaining upper-extremity function and promoting functional use of the affected hand. During the postoperative rehabilitation period, the patient received analgesic medications for postoperative pain management as needed. No medications specifically targeting neurological recovery or nerve regeneration were administered.

### 2.2. Metacarpophalangeal Joint Training Using the Single-Joint HAL

We developed a novel HAL-based training system (MP-HAL) for flexion and extension training of the MP joints of the left hand in this patient by applying the single-joint type of the HAL [14]. The MP-HAL system consists of an actuator, a support base, a connecting frame, and a finger attachment (Figure 5).

One arm of the actuator was fixed to the support base (Figure 5, yellow arrowheads) and placed stably on a training table. Subsequently, the connecting frame was attached to the other arm of the actuator (Figure 5, orange arrowhead), and the finger attachment was mounted onto the connecting frame.

The patient inserted the second to fifth digits of the left hand into the finger attachment, thereby securing the fingers in place. The forearm was then positioned on the table such that the MP joints of the second to fifth digits were aligned with the rotational axis of the actuator. During training, the wrist was immobilized using a wrist splint to prevent unintended motion.

Surface electrode sensors of the HAL were placed on the skin over the palmar intrinsic hand muscles (lumbricals) for MP joint flexion and over the extensor digitorum muscle for MP joint extension.

The patient was seated and performed flexion and extension training of the MP joints with the left forearm placed on a padded mat positioned on the table (Figure 5) (Video S2). During training, the therapist appropriately adjusted the level of HAL assistance as well as the maximum flexion and extension angles of the patient’s MP joints.

### 2.3. Wrist Abduction Training Using the Single-Joint HAL

Following MP-HAL training, we performed wrist abduction training toward the ulnar side of the left hand in this patient using the single-joint type of the HAL (Wrist-Abd HAL).

The support base used in the MP-HAL system was also utilized to fix one arm of the actuator of the single-joint HAL. At that time, the actuator was positioned so that its rotational direction corresponded to the direction of ulnar deviation of the patient’s wrist. The forearm was placed on the table such that the center of the wrist joint was aligned with the rotational axis of the actuator (Figure 6).

The distal portion of the patient’s forearm and hand was secured to the other arm of the actuator, and wrist abduction training toward the ulnar side (Wrist-Abd HAL training) was performed (Video S3). Surface electrode sensors of HAL were placed on the skin over the extensor carpi ulnaris muscle.

### 2.4. Implementation of HAL Training

The setup of the MP-HAL and Wrist-Abd HAL required approximately 3 min. The actual training time was about 20 min each for MP joint flexion–extension and for ulnar deviation of the wrist. The training duration was determined with reference to our previously reported HAL rehabilitation protocols for postoperative upper-extremity paralysis. Whereas shoulder HAL training was typically performed for 30–45 min and elbow HAL training for approximately 15–20 min, MP-HAL and Wrist-Abd HAL training sessions were each conducted for approximately 20 min, considering the smaller target muscle groups and the potential for fatigue. In accordance with previous HAL studies, assistance levels were individually adjusted based on the patient’s fatigue, comfort, pain, and quality of movement during each session [18].

The patient began MP-HAL training on postoperative day 3, with the plan to switch to Wrist-Abd HAL training at an appropriate time. The operating conditions of the MP-HAL and Wrist-Abd HAL, as set by the therapist, were generally as follows: Cybernic Voluntary Control–Gentle mode; Torque Limit: 10–30; Bioelectrical Signal-based Balance Control: 100% flexion and 30% extension. Based on the patient’s feedback regarding comfort and smoothness of movement, the physical therapist or physician supervised the training and monitored the device during each session.

Throughout the intervention period, adverse events were clinically monitored by the supervising therapist and physician at each training session. Particular attention was paid to pain exacerbation, excessive fatigue, skin irritation at electrode or attachment sites, device-related discomfort, and neurological worsening. Pain intensity was assessed using a visual analog scale (VAS).

The interval between HAL sessions varied during the intervention period because the patient transitioned from inpatient to outpatient rehabilitation. During hospitalization, HAL sessions were performed more frequently, whereas after discharge the interval between sessions increased according to the outpatient rehabilitation schedule. This approach was consistent with our previously reported HAL rehabilitation protocol for postoperative upper-extremity motor deficits [18]. The timing and frequency of HAL training were therefore determined primarily by the clinical rehabilitation setting and patient availability rather than by predefined criteria based on treatment response.

### 2.5. Morphological and Functional Evaluation

Morphological and functional evaluations, including the maximum active extension angle of the MP joints, grip strength, and lateral pinch strength, were performed before each MP-HAL training session. In addition, patient-reported upper-extremity disability was assessed using the Quick Disabilities of the Arm, Shoulder and Hand (QuickDASH) questionnaire at Pre MP-HAL intervention, after 10 MP-HAL sessions, after completion of the MP-HAL intervention, and after completion of the Wrist-Abd HAL intervention. During the subsequent Wrist-Abd HAL intervention period, clinical changes in wrist alignment and finger posture were documented photographically (Figure 4).

### 2.6. Surface Electromyography Analysis

Muscle activity of the left extensor digitorum communis and the left lumbrical muscles was recorded using the Trigno™ Lab Wireless Surface EMG system (Delsys Inc., Boston, MA, USA). EMG signals were sampled at 2000 Hz. Surface electromyography measurements were performed during the 3rd, 4th, and 14th MP-HAL training sessions.

Muscle activity was recorded during MP joint flexion and extension tasks under both no-HAL and HAL-assisted conditions. Representative EMG recordings obtained during the 3rd, 4th, and 14th sessions were selected for presentation to illustrate changes in muscle activation patterns over the course of training. The EMG waveforms were visually compared between the no-HAL and HAL-assisted conditions to evaluate activation timing and overlap between the extensor digitorum communis and lumbrical muscles. Because the purpose of the EMG assessment was to qualitatively evaluate muscle activation patterns and co-contraction, no quantitative EMG variables or normalization procedures were applied.

EMG electrodes were placed over the extensor digitorum communis, extensor carpi ulnaris, and lumbrical muscles based on anatomical landmarks corresponding to previously described motor point locations and intramuscular nerve distributions. The placement strategy was informed by ultrasound-based anatomical mapping of distal upper limb muscles reported in recent literature [19], ensuring reliable recording from functionally relevant muscle regions.

To quantitatively evaluate wrist kinematics and muscle activity during active wrist ulnar flexion, a three-dimensional motion capture system (Vicon Motion Systems Ltd., Oxford, UK) and surface electromyography were used. Assessments were performed under both no-HAL and HAL-assisted conditions immediately before the initiation of Wrist-Abd HAL training (Pre; prior to Session 22) and after completion of the Wrist-Abd HAL intervention (Post; after Session 26). Reflective markers were placed on the lateral aspect of the forearm at approximately 50% of forearm length, the radial styloid process, the ulnar styloid process, the dorsal aspect of the distal metacarpal region, and the distal phalanges of the second and fifth digits. Wrist radial/ulnar deviation angles were calculated from the relative orientation of the forearm and hand segments defined by these anatomical landmarks. Surface electromyography of the left flexor carpi ulnaris was simultaneously recorded using the Trigno™ Lab Wireless Surface EMG system (Delsys Inc., Boston, MA, USA). Wrist kinematics and flexor carpi ulnaris muscle activity obtained under the no-HAL and HAL-assisted conditions were compared to evaluate changes associated with Wrist-Abd HAL training.

## 3. Results

In this case, bioelectrical signals could be detected from the palmar intrinsic muscles (lumbricals) and the extensor digitorum communis, enabling voluntary flexion and extension of the MP joints with the HAL. In addition, voluntary ulnar deviation training of the wrist using bioelectrical signals from the extensor carpi ulnaris was also feasible. A total of 27 HAL training sessions were conducted (21 sessions of MP-HAL and 6 sessions of Wrist-Abd HAL) (Figure 7). No device-related adverse events, excessive fatigue, skin complications, or neurological deterioration were observed during the intervention period. Pain intensity remained 0 on the VAS throughout all training sessions.

MP-HAL training was initiated on postoperative day 3 and was performed three times per week during the inpatient period until postoperative day 14 (Figure 4). After postoperative day 14, training was continued on an outpatient basis, as follows: two to four times per month until 3.5 months postoperatively, and once per month from 3.5 months to 1 year postoperatively. A total of 21 MP-HAL training sessions were conducted (Figure 7).

From the 22nd session onward, training was switched to Wrist-Abd HAL (Figure 4). Wrist-Abd HAL training was performed once per month after 1 year postoperatively. A total of six Wrist-Abd HAL sessions were conducted from the 22nd to the 27th session (Figure 7).

The number of MP joint flexion–extension repetitions during MP-HAL training was 100 in the first session, gradually increased, and reached 300 by the 12th session. From the 12th to the 21st session, 300 repetitions were performed (Figure 7). The HAL assist gain level was gradually increased after the first session and reached 40, providing substantial assistance, by the 7th session. Thereafter, even when the assist gain level was reduced, flexion–extension movements could be performed smoothly. From the 15th session onward, the assist gain level was decreased to 5 (Figure 7), indicating that smooth flexion–extension movements could be performed voluntarily with minimal assistance.

With MP-HAL training, the maximum extension angle of the MP joints of the left index to little fingers gradually increased after the first session, and by the 21st session extension to 0° was achieved in all fingers except the ring finger, resulting in marked improvement in finger drop (Figure 8). In the functional evaluation, comparison between the first and 21st sessions showed that grip strength increased from 12 kg to 20 kg, and lateral pinch strength increased from 3.0 kg to 8.0 kg (Figure 9). However, radial deviation of the wrist during finger abduction and extension persisted in the left hand (Figure 10, arrowhead) (Video S4).

Therefore, using the Wrist-Abd HAL system, 200 repetitions of ulnar deviation training of the wrist were performed in each session from the 22nd to the 27th session (Figure 7). The HAL assist gain level was set between 5 and 10, allowing ulnar deviation training to be performed with minimal assistance (Figure 7). With Wrist-Abd HAL training, the radial deviation of the wrist gradually improved, and by the 27th session radial deviation during finger abduction and extension was scarcely observed (Figure 11) (Video S5).

Representative EMG recordings obtained during the 3rd (Figure 12a), 4th (Figure 12b), and 14th (Figure 12c) MP-HAL training sessions are shown in Figure 12. Gray bands indicate periods of extensor digitorum communis activation; the same time windows are displayed for the lumbrical muscle to facilitate comparison of activation timing between muscles. In Session 3 (Figure 12a), muscle activity of both the extensor digitorum communis and lumbrical muscles was relatively weak under the no-HAL condition. During HAL-assisted movement, activation of both muscles increased. Furthermore, lumbrical muscle activity appeared less synchronized with extensor activation, suggesting reduced co-contraction and improved separation of muscle activation patterns. In Session 4 (Figure 12b), muscle activation was observed in both muscles under the no-HAL condition, indicating improved activation compared with Session 3. However, substantial overlap between extensor and lumbrical muscle activity remained within the extensor activation periods, suggesting persistent co-contraction. Under the HAL-assisted condition, overlap between the two muscles appeared reduced, with a clearer temporal separation of muscle activity. In Session 14 (Figure 12c), muscle activation patterns under the no-HAL condition were further improved compared with Session 3; however, co-contraction remained evident, as lumbrical muscle activity continued to overlap with extensor activation periods. During HAL-assisted movement, muscle activity appeared more selectively timed, with reduced overlap between the extensor digitorum communis and lumbrical muscles, suggesting enhanced dissociation of muscle activation.

Three-dimensional motion analysis and surface electromyography during active wrist ulnar flexion are shown in Figure 13. At the Pre assessment, the mean wrist ulnar flexion angle was greater under the HAL-assisted condition than under the no-HAL condition (44° vs. 20°, respectively; Figure 13a). Representative recordings also demonstrated greater flexor carpi ulnaris muscle activity and a larger excursion of wrist motion during HAL-assisted movement (Figure 13b). At the Post assessment, the mean wrist ulnar flexion angle under the no-HAL condition increased compared with the Pre assessment (31° vs. 20°), whereas the difference between the no-HAL and HAL-assisted conditions became smaller (31° vs. 34°, respectively; Figure 13c). Representative recordings showed preserved wrist movement under both conditions, although flexor carpi ulnaris activity remained greater during HAL-assisted movement (Figure 13d).

The QuickDASH score decreased from 45.0 at Pre MP-HAL intervention to 15.9 after 10 MP-HAL sessions, 9.1 after completion of the MP-HAL intervention, and 4.5 after completion of the Wrist-Abd HAL intervention (Table 1).

## 4. Discussion

Previous studies have reported that drop finger can occur in patients with cervical spondylotic radiculopathy or cervical spondylotic amyotrophy [1,2,3,4,5,6]. In the present case, drop finger developed as a result of cervical spondylotic radiculopathy, and imaging studies demonstrated compression of the left C7 and C8 nerve roots. Drop finger is caused by weakness of extension at the MP joints. The principal muscle responsible for MP joint extension is the extensor digitorum, which is innervated predominantly by the C7 nerve root, with additional contribution from C8 [18]. In the early stage of symptom onset, our patient exhibited weakness of the left triceps brachii muscle, a finding typically associated with C7 radiculopathy. These findings suggest that C7 impairment contributed to the development of drop finger in this case. In addition, marked weakness of the intrinsic hand muscles, including the interossei and lumbricals, was observed in the left hand. These intrinsic muscles are primarily innervated by the C8–T1 nerve roots [20]. Furthermore, sensory disturbance was clearly present in the left C8 dermatome. These findings indicate that C8 impairment also substantially contributed to the neurological deficits in this patient. Taken together, we consider that the drop finger in the present case was caused by combined impairment of both the C7 and C8 nerve roots.

In addition to drop finger, the present patient exhibited radial deviation of the left wrist. To the best of our knowledge, there have been no previous reports describing radial deviation of the wrist in patients with cervical spondylotic radiculopathy. In contrast, radial deviation of the wrist has been reported in patients with posterior interosseous nerve palsy, a peripheral nerve disorder [21]. In posterior interosseous nerve palsy, the extensor carpi ulnaris (ECU) is paralyzed, whereas the extensor carpi radialis longus (ECRL) is preserved. This imbalance among the muscles controlling wrist movement is considered to result in radial deviation of the wrist. A similar mechanism may explain the findings in the present case. Myelographic findings (Figure 2) demonstrated foraminal lesions at the left C6–7 and C7–T1 levels, corresponding to C7 and C8 nerve root compression. The ECRL is the muscle most strongly involved in wrist extension and is innervated predominantly by the C6 nerve root, with additional contribution from C7 [20]. Therefore, impairment of the ECRL was considered relatively mild in the present case. In contrast, the ECU, which also contributes to wrist stabilization, is innervated by the C7–C8 nerve roots [20], and may have been more severely affected. We therefore speculate that the radial deviation of the wrist observed in the present patient resulted from an imbalance between radial- and ulnar-sided wrist extensor muscles.

Conservative treatment is generally considered the first-line therapy for cervical radiculopathy, and many patients improve within several weeks to months [22,23]. However, in patients with drop finger caused by cervical spondylotic radiculopathy, conservative treatment is often ineffective, and early surgical intervention has been recommended [1,3,4]. Surgical treatment for drop finger typically consists of decompression of the affected nerve roots; nevertheless, postoperative outcomes are not always satisfactory [7,8]. Delayed surgical intervention has been associated with poorer recovery, particularly when surgery is performed more than 6 months after symptom onset [5]. In addition, severe preoperative drop finger has been reported to correlate with poor postoperative recovery [2,3,6].

In the present case, surgical treatment was selected 3 months after the onset of drop finger. Left-sided C6–7 and C7–T1 foraminotomies were performed to achieve posterior decompression of the left C7 and C8 nerve roots. In addition, a herniated disc located in the left C7–T1 foramen was removed. Based on previous reports describing surgical outcomes of drop finger associated with cervical spondylotic radiculopathy, insufficient postoperative recovery of drop finger was anticipated in the present case. Therefore, an additional strategy to facilitate recovery of drop finger was considered necessary.

To the best of our knowledge, there have been no reports describing postoperative rehabilitation for drop finger caused by cervical spondylotic radiculopathy in detail. In current clinical practice, postoperative rehabilitation is generally performed according to treatment strategies used for peripheral nerve injuries of the upper extremity [11,12]. These conventional rehabilitation approaches include splinting or orthotic therapy to assist MP joint extension, prevention of joint contracture and maintenance of range of motion, and training in functional hand use while wearing orthoses [10]. However, these traditional rehabilitation strategies for drop finger are not intended to restore the lost muscle strength itself, that is, they are not designed to directly improve the drop finger. Taken together, conventional postoperative rehabilitation appears to have clear limitations in achieving meaningful recovery of muscle strength in patients with drop finger.

In the present case, we performed HAL-based rehabilitation from the early postoperative stage with the specific aim of improving the drop finger itself. A key consideration in developing MP-HAL training was determining the optimal source of bioelectrical signals for triggering the device. We selected bioelectrical signals from the extensor digitorum muscle to trigger MP joint extension and signals from the lumbrical muscles, which function as intrinsic hand muscles, to trigger MP joint flexion. The anatomical appropriateness of these recording sites is supported by recent ultrasound-based studies describing motor point localization and intramuscular nerve distribution in distal upper-limb muscles [19]. This anatomical framework may enhance the interpretability of the electromyographic findings obtained in the present case. As a result, voluntary motor training for flexion and extension of the MP joints could be performed smoothly using this method. To the best of our knowledge, there have been no previous reports describing voluntary motor training specifically targeting flexion and extension movements of the MP joints in the field of rehabilitation, making this the first such report. Ultimately, the patient regained normal active motion of the MP joints, and the drop finger completely resolved.

At our institution, clinical trials of HAL-based functional recovery therapy have been conducted for various orthopedic disorders that are difficult to treat using conventional rehabilitation alone. In patients with severe spinal cord disorders presenting with lower-extremity paralysis, gait training using the bilateral leg HAL improved gait speed, stride length, and cadence [24]. In addition, HAL training was shown to suppress co-contraction between the extensor and flexor muscles of the lower extremities, resulting in a reduction in lower-limb spasticity. Patients with severe spinal cord disorders commonly exhibit marked disturbances in muscle synergies of the lower extremities; engineering analyses have demonstrated that HAL training can normalize these abnormal muscle synergies [25].

We previously performed rehabilitation using the single-joint HAL following intercostal nerve transfer for brachial plexus injury, with the aim of achieving early recovery of elbow flexion function [26]. The use of HAL enabled the initiation of voluntary elbow flexion training during the early postoperative period. Furthermore, HAL training facilitated dissociation between the elbow flexor muscles, particularly the biceps brachii, and the elbow extensor muscles, namely the triceps brachii.

We also performed voluntary shoulder abduction training using the single-joint HAL from the early postoperative period in patients with postoperative deltoid paralysis following cervical spine surgery (so-called C5 palsy), and achieved early improvement in the paralysis [27]. Furthermore, engineering analyses demonstrated that shoulder HAL training suppressed abnormal compensatory movements mediated by the trapezius muscle associated with C5 palsy, thereby restoring a more physiological shoulder movement pattern [28]. In addition, we previously reported a chronic case in which conventional rehabilitation had failed to improve C5 palsy and severe paralysis persisted for 41 months after surgery; following shoulder HAL training, the patient regained the ability to abduct the shoulder joint [18].

In the present case, the use of HAL enabled voluntary flexion and extension training of the MP joints within the normal range of motion from the early phase of rehabilitation. Furthermore, electromyographic examination demonstrated clearly detectable muscle activity even during the initial stage of MP-HAL training. These findings suggest that, even in the early postoperative phase when neurological recovery remained minimal, HAL enabled the patient to repeatedly perform voluntary flexion and extension movements of the MP joints within a physiological range of motion. Such training may have promoted the reacquisition of coordinated movement patterns and contributed to the early improvement in MP joint function, although the underlying neural mechanisms remain unclear.

This mechanism may have contributed to the early improvement in MP joint range of motion. Indeed, compared with previous reports, improvement in drop finger was achieved relatively early in the present case [3]. In addition, electromyographic findings demonstrated dissociation between the extensor and flexor muscles following HAL training. The electromyographic findings suggest that the intended target muscles actively contributed to the training movements. However, because electromyographic assessment was limited to the extensor digitorum communis and lumbrical muscles, the contribution of other compensatory muscles cannot be completely excluded.

The EMG recordings demonstrated that muscle activation of both the extensor digitorum communis and lumbrical muscles increased over time under both the no-HAL and HAL-assisted conditions. Because this patient was also recovering from surgery during the intervention period, it is difficult to distinguish the extent to which the increased muscle activity was attributable to natural postoperative recovery or to the effects of HAL training. However, reduced overlap between extensor and lumbrical muscle activity was consistently observed only during HAL-assisted movement across Sessions 3, 4, and 14. In contrast, co-contraction remained evident under the no-HAL condition, even in the later sessions. These findings suggest that HAL assistance may have facilitated more selective muscle activation and improved intermuscular coordination by promoting dissociated activation of the extensor and flexor muscles during task performance. Notably, the improved separation of muscle activity observed during HAL-assisted movement was not clearly maintained under the no-HAL condition by Session 14. This finding may indicate that motor relearning was still in progress and that the newly acquired movement pattern had not yet been fully retained or generalized to voluntary movement without HAL assistance. Therefore, repeated HAL-assisted practice may have contributed to the process of motor learning, although the persistence of these effects after device removal requires further investigation.

The kinematic analysis demonstrated a marked increase in wrist ulnar flexion range of motion during HAL-assisted movement before initiation of Wrist-Abd HAL training. In contrast, after completion of the intervention, wrist ulnar flexion under the no-HAL condition had improved and the difference between conditions became smaller. These findings may suggest that movement patterns facilitated by HAL assistance were partially retained and expressed during voluntary movement without the device. Furthermore, increased flexor carpi ulnaris activity observed during HAL-assisted movement may indicate that HAL promoted activation of muscles responsible for wrist stabilization and ulnar flexion. However, because this was a single-case observation, the mechanisms underlying these changes remain speculative and require confirmation in future studies.

Similar to the effects previously reported in patients with severe spinal cord disorders and postoperative brachial plexus injury [25,26], this finding may reflect suppression of abnormal co-contraction induced by HAL training. In other words, the therapeutic effect of MP-HAL training may not simply represent recovery of muscle strength, but rather reacquisition of motor control between agonist and antagonist muscles.

The precise mechanism underlying the observed recovery remains unclear. HAL does not directly promote nerve regeneration or reinnervation; rather, it provides movement assistance synchronized with the patient’s voluntary muscle activity, enabling repeated execution of intended movements accompanied by sensory feedback. This process may reinforce the sensorimotor loop and facilitate motor relearning during recovery. However, spontaneous neurological recovery following surgical decompression cannot be excluded. Therefore, the relative contribution of HAL training, conventional rehabilitation, surgical decompression, and natural postoperative recovery to the observed functional improvement cannot be determined in the present case. Furthermore, no neuroimaging or neurophysiological assessments were performed; therefore, cortical reorganization and other neural mechanisms cannot be verified and remain speculative.

Previous reports have described successful outcomes of tendon transfer surgery for radial deviation of the wrist [29]. However, to the best of our knowledge, there have been no reports describing improvement in radial deviation of the wrist using a non-surgical rehabilitation approach. In the present case, in addition to MP-HAL training, we developed Wrist-Abd HAL training to facilitate voluntary ulnar-direction wrist abduction movements. To our knowledge, there have been no previous reports describing voluntary wrist abduction training using this approach, making this the first report of its kind. The therapeutic effects of Wrist-Abd HAL training may be explained in a manner similar to that of MP-HAL training. Even at a stage when recovery from paralysis remained insufficient, the use of HAL enabled the patient to perform voluntary wrist movements within the normal range of motion. This successful motor experience may have facilitated the reacquisition of normal wrist movement patterns.

This study has several limitations. First, this is a single-case report, and therefore the findings should be interpreted with caution. The observed recovery cannot be generalized to other patients with drop finger caused by cervical radiculopathy. Second, no control condition was available, and the patient concurrently underwent conventional rehabilitation following surgical decompression. Therefore, the relative contributions of HAL training, conventional rehabilitation, and natural postoperative recovery cannot be determined. Third, nerve conduction studies and needle electromyography were not performed. Consequently, objective electrophysiological confirmation of the affected nerve roots and the extent of denervation was unavailable. Fourth, outcome assessments were not blinded, which may have introduced assessment bias. Fifth, although wrist radial/ulnar deviation was quantitatively assessed using motion analysis, the kinematic findings were obtained from a single patient and should be interpreted with caution. Furthermore, repeated measurements and comparisons with normative data were not performed. Finally, the underlying mechanisms of recovery remain speculative. Although repetitive sensorimotor training with HAL may have facilitated motor relearning and improved muscle coordination, this should be considered a possible explanation rather than a definitive conclusion. Further studies involving larger cohorts and controlled study designs are required to confirm the present findings.

## 5. Conclusions

In summary, we report a patient with drop finger and radial deviation of the wrist caused by C7 and C8 cervical radiculopathy due to cervical spondylotic disease who underwent postoperative rehabilitation using novel HAL-based training programs (MP-HAL training and Wrist-Abd HAL training) initiated in the early postoperative period following nerve root decompression surgery. During the clinical course, improvement in both drop finger and radial deviation of the wrist was observed. The use of HAL enabled voluntary motor training of the fingers and wrist within the normal range of motion even during the early postoperative phase, when neurological recovery remained insufficient. This experience of successful voluntary movement may have contributed to the reacquisition of more physiological movement patterns. However, given the concurrent effects of surgical decompression, conventional rehabilitation, and natural postoperative recovery, the contribution of HAL training to the observed improvement cannot be determined in this single-case report. Further accumulation of cases is required to clarify the potential role of HAL-based rehabilitation as a therapeutic option for drop finger and wrist radial deviation.

## Figures and Tables

**Figure 1:**
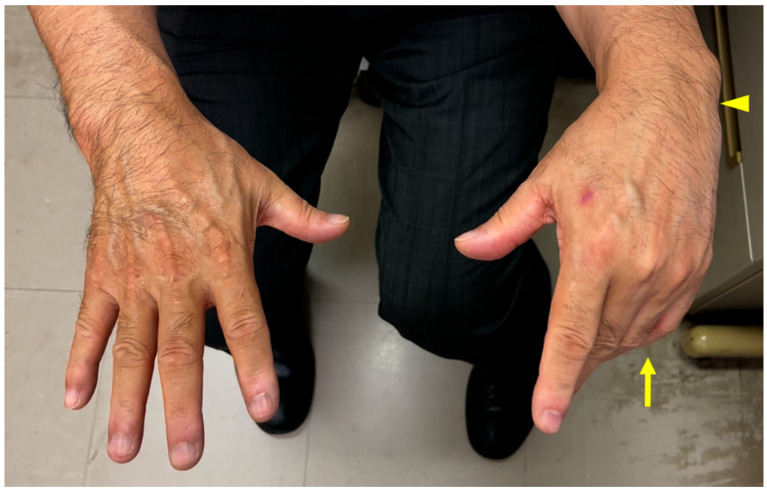
Preoperative findings in the bilateral hands in the extended and abducted position. The left hand shows drop finger.

**Figure 2:**
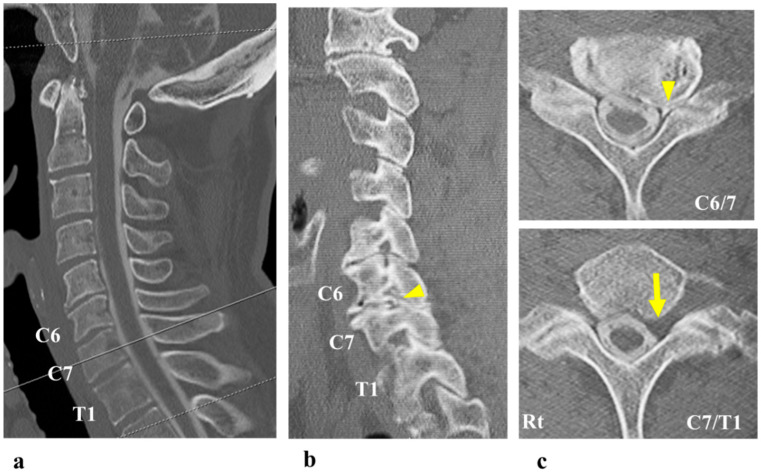
Preoperative cervical CT images after myelography. (**a**) Midsagittal reconstructed image showing no apparent spinal cord compression. (**b**) Left parasagittal reconstructed image along the C6–7 and C7–T1 foramina, demonstrating an osteophyte within the left C6–7 foramen (arrowhead). (**c**) Axial images at the levels of C6–7 and C7–T1, showing an osteophyte within the left C6–7 foramen (arrowhead) and a space-occupying lesion suggestive of a herniated disc within the left C7–T1 foramen (arrow).

**Figure 3:**
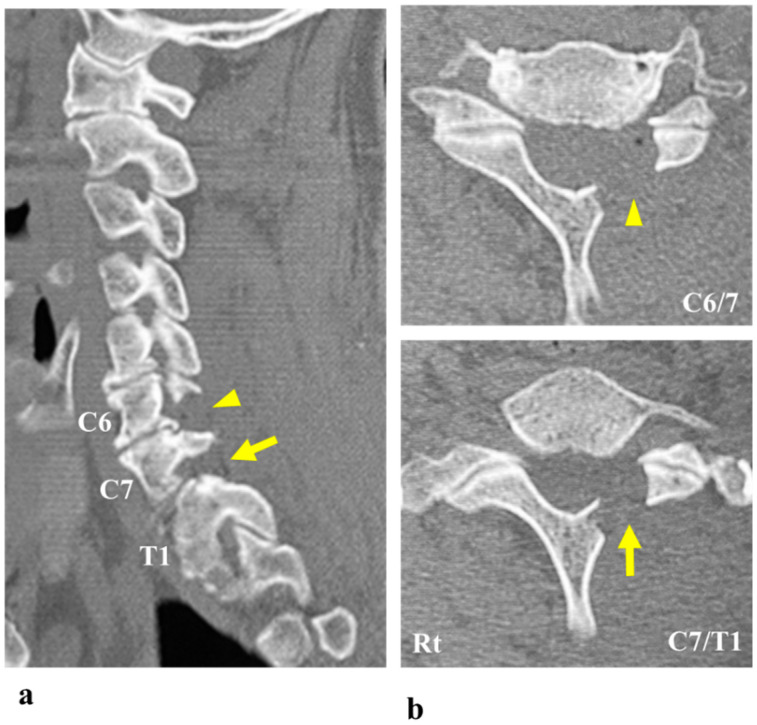
Postoperative cervical CT images. (**a**) Left parasagittal reconstructed image along the C6–7 and C7–T1 foramina, showing adequate left C6–7 foraminotomy (arrowhead) and C7–T1 foraminotomy (arrow). (**b**) Axial images at the levels of C6–7 and C7–T1, also demonstrating adequate left C6–7 foraminotomy (arrowhead) and C7–T1 foraminotomy (arrow).

**Figure 4:**
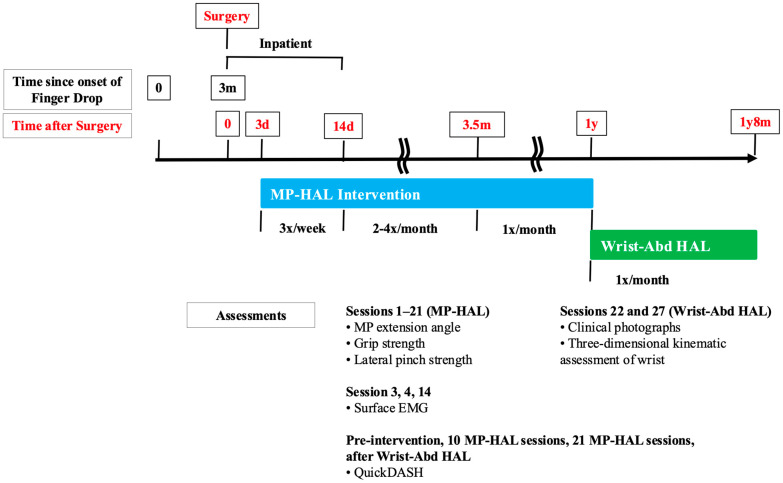
Clinical timeline after the onset of drop finger, showing surgical intervention, postoperative Hybrid Assistive Limb (HAL) training, and the timing of clinical and electromyographic assessments.

**Figure 5:**
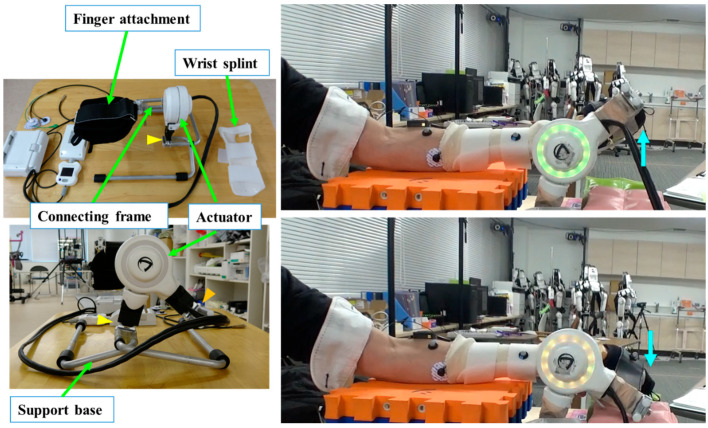
Structure of the MP HAL and training scenes of MP joint flexion and extension in this case. (**Left**): One arm of the actuator is attached to the support base (yellow arrowheads). The connecting frame with the finger attachment is mounted on the other arm of the actuator (orange arrowhead). (**Right**): The index to little fingers of the left hand are inserted into and secured with the finger attachment. The left hand is positioned so that the MP joints of digits 2–5 align with the rotational axis of the actuator. Wrist motion is restricted with a wrist splint. Training of MP extension (**right upper**) and MP flexion (**right lower**).

**Figure 6:**
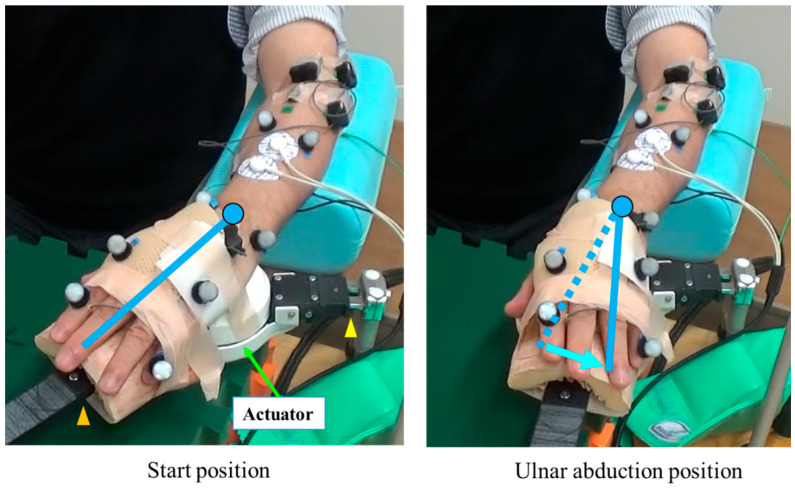
Ulnar deviation training of the left wrist using the Wrist-Abd HAL. One arm of the actuator is fixed to the support base (yellow arrowhead). The actuator is positioned so that its rotational direction is aligned with the direction of ulnar deviation of the patient’s wrist. The distal part of the patient’s wrist is secured to the other arm of the actuator (orange arrowhead) to perform ulnar deviation training of the wrist (Wrist-Abd HAL training).

**Figure 7:**
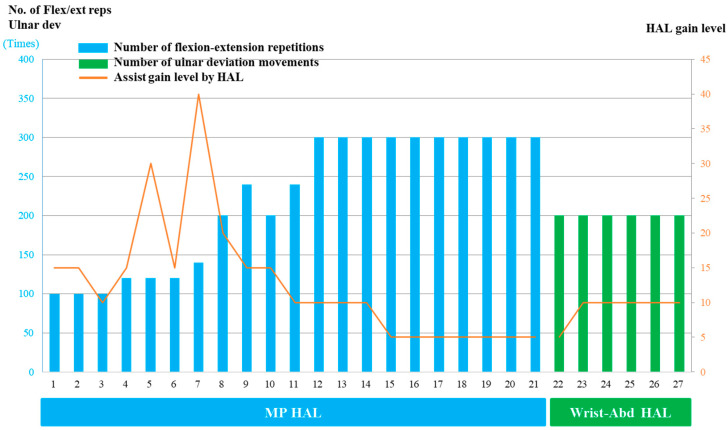
Number of MP joint flexion–extension and wrist ulnar deviation movements and HAL assist gain level in each session of MP HAL and Wrist-Abd HAL training.

**Figure 8:**
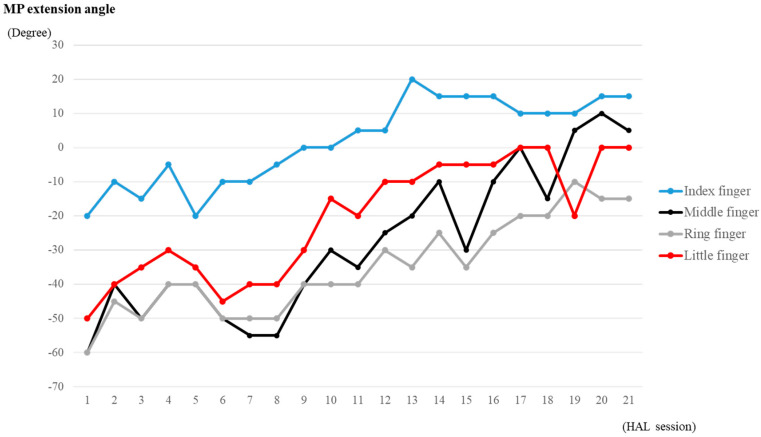
Changes in the maximum active extension angle of the MP joints of the left index to little fingers during the MP HAL training period.

**Figure 9:**
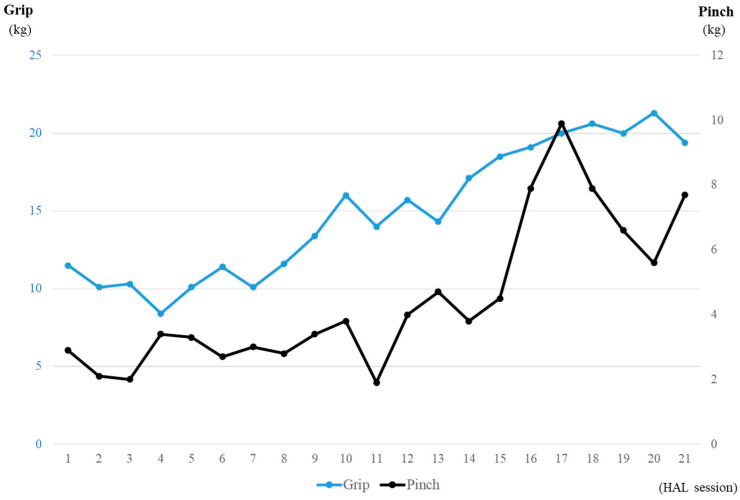
Changes in grip strength and lateral pinch strength of the left hand during the MP-HAL training period.

**Figure 10:**
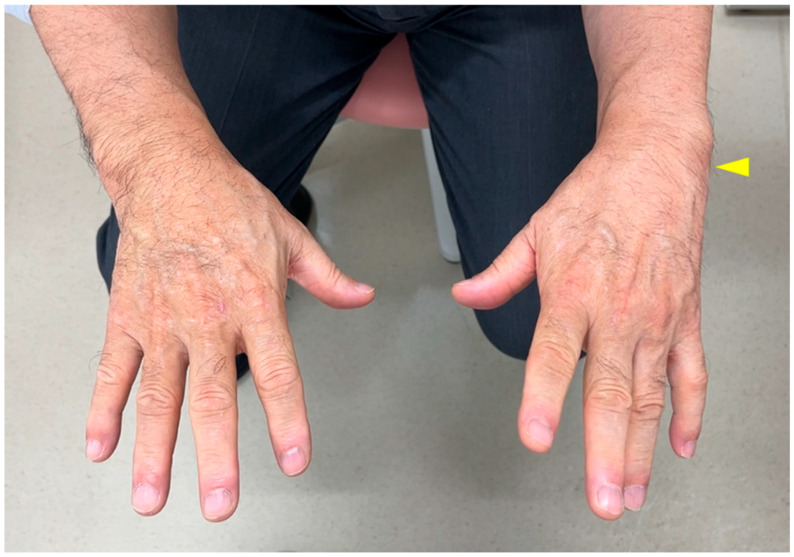
Findings of both hands in the abducted position at the 21st session of MP HAL training. In the left hand, extension to 0° was achieved in all fingers except the ring finger, and drop finger was markedly improved. However, radial deviation of the wrist during finger abduction and extension persisted.

**Figure 11:**
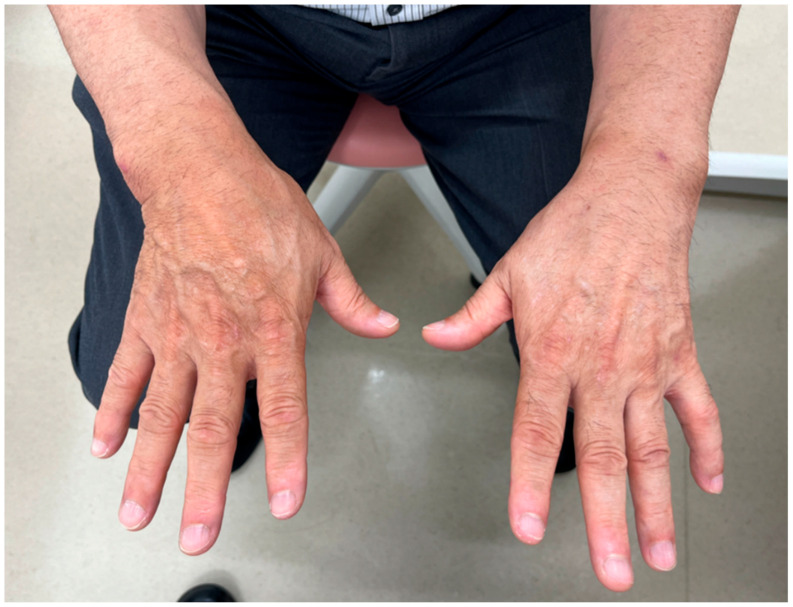
Findings of both hands in the abducted position at the 27th session of Wrist-Abd HAL training. Radial deviation of the wrist during finger abduction and extension was scarcely observed.

**Figure 12:**
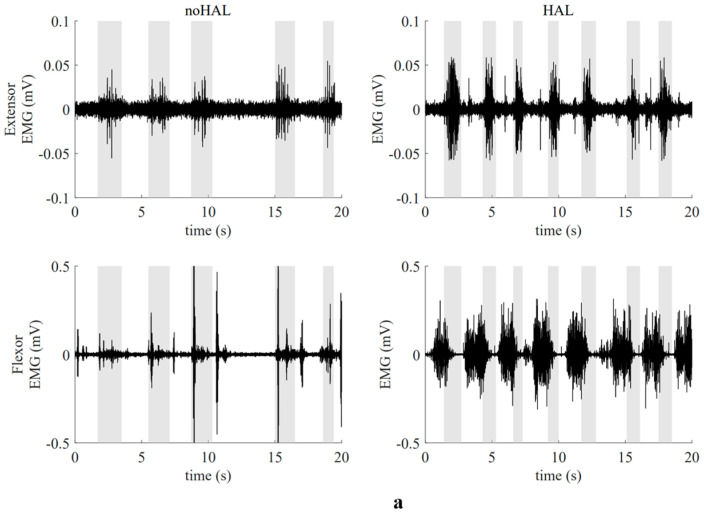

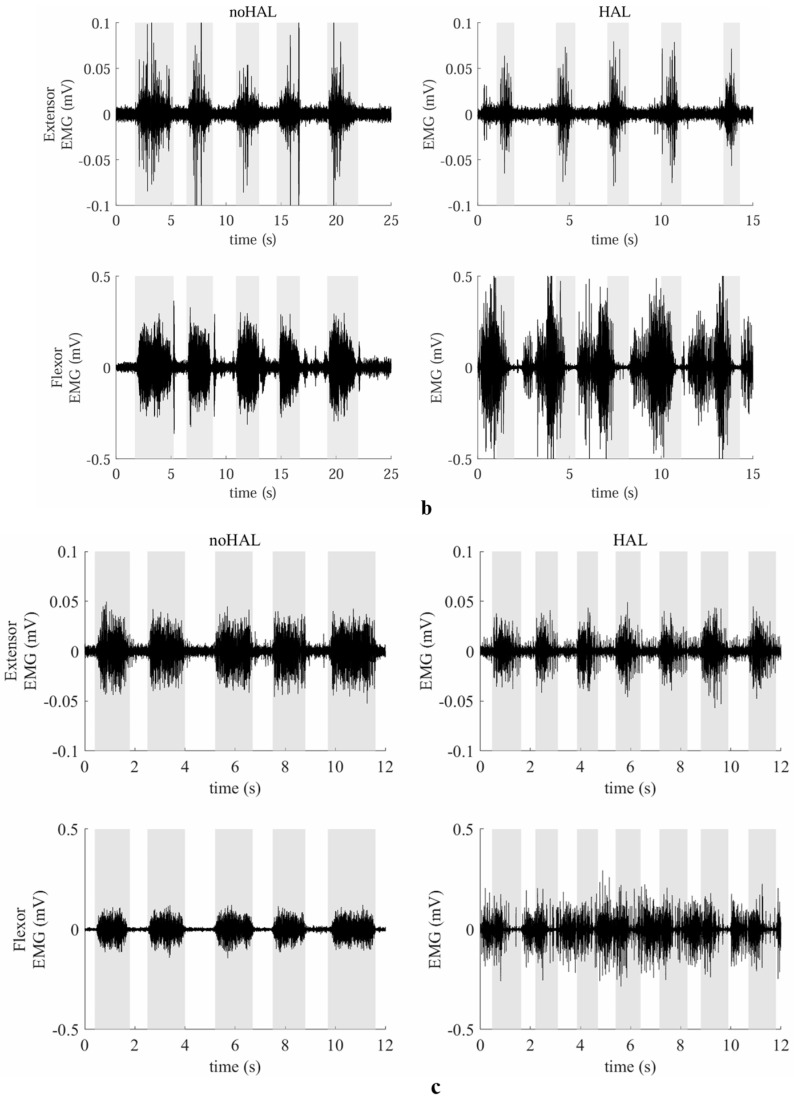
Representative surface electromyogram recordings of the left extensor digitorum communis and lumbrical muscles obtained during the 3rd (**a**), 4th (**b**), and 14th (**c**) MP-HAL training sessions under no-HAL and HAL-assisted conditions. Gray bands indicate periods of extensor digitorum communis activation (MP extension phase). The same time windows are displayed for the lumbrical muscle to facilitate comparison of activation timing between muscles. In Sessions 4 and 14, muscle activation under the no-HAL condition appeared greater than that observed in Session 3; however, overlap between extensor digitorum communis and lumbrical muscle activity remained evident. Under the HAL-assisted condition, overlap between the two muscles appeared reduced, with clearer temporal separation of muscle activity across all sessions.

**Figure 13:**
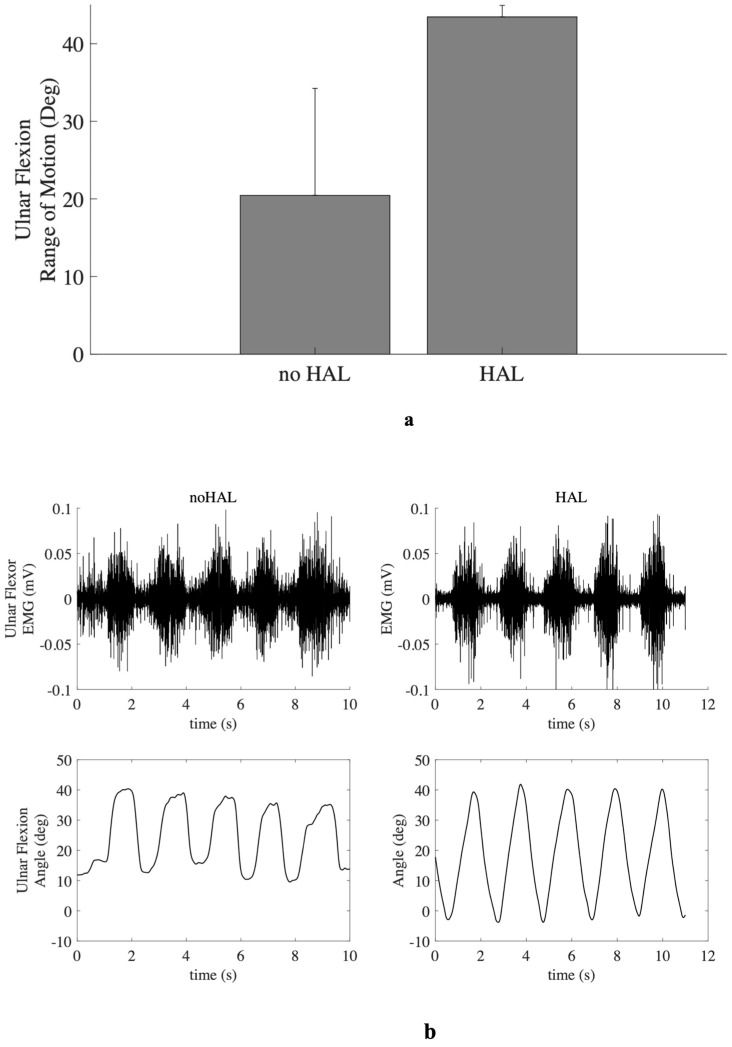

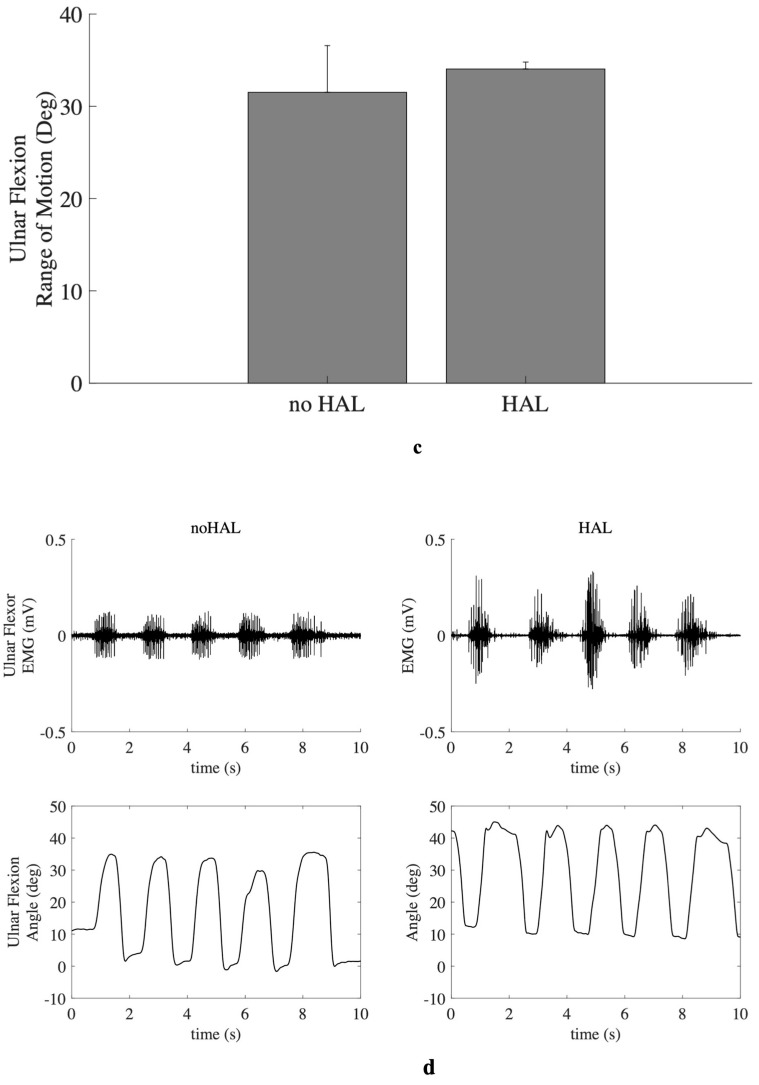
Kinematic and electromyographic assessment during active wrist ulnar flexion before and after Wrist-Abd HAL training. (**a**) Mean wrist ulnar flexion angle obtained from five trials under no-HAL and HAL-assisted conditions before initiation of Wrist-Abd HAL training (Pre). (**b**) Representative recordings of left flexor carpi ulnaris electromyographic activity and wrist ulnar flexion angle during the Pre assessment. (**c**) Mean wrist ulnar flexion angle obtained from five trials under no-HAL and HAL-assisted conditions after completion of Wrist-Abd HAL training (Post). (**d**) Representative recordings of left flexor carpi ulnaris electromyographic activity and wrist ulnar flexion angle during the Post assessment. Pre indicates the assessment performed immediately before Wrist-Abd HAL training (prior to Session 22), and Post indicates the assessment performed after completion of Wrist-Abd HAL training (after Session 26).

**Table 1:** Changes in QuickDASH scores during the intervention period.

Assessment Point	Total Score	Number of Completed Items	QuickDASH Score
Pre 1 MP-HAL sessions	28	10	45.0
After 10 MP-HAL sessions	18	11	15.9
After completion of MP-HAL intervention (Session 21)	15	11	9.1
After completion of Wrist-Abd HAL intervention	13	11	4.5

## Data Availability

The original contributions presented in this study are included in the article. Further inquiries can be directed to the corresponding author.

## Supplementary Materials

The following supporting information can be downloaded at: https://www.mdpi.com/article/10.3390/jcm15135182/s1, Video S1: Bilateral finger abduction movements before surgery; Video S2: Training of left metacarpophalangeal joint extension and flexion movements using the MP HAL; Video S3: Training of left wrist ulnar-direction abduction movements using the Wrist-Abd HAL; Video S4: Bilateral finger abduction movements after completion of the 21st HAL training session; Video S5: Bilateral finger abduction movements after completion of the 27th HAL training session.
